# EEG Global Coherence in Scholar ADHD Children during Visual Object Processing

**DOI:** 10.3390/ijerph19105953

**Published:** 2022-05-13

**Authors:** Loyda Hernández-Andrade, Ana Cristina Hermosillo-Abundis, Brenda Lesly Betancourt-Navarrete, Diane Ruge, Carlos Trenado, Rafael Lemuz-López, Héctor Juan Pelayo-González, Vicente Arturo López-Cortés, María del Rosario Bonilla-Sánchez, Marco Antonio García-Flores, Ignacio Méndez-Balbuena

**Affiliations:** 1 Facultad de Psicología, Benemérita Universidad Autónoma de Puebla, Puebla 72000, Mexico; loydah.andrade@gmail.com (L.H.-A.); betancourtbl@gmail.com (B.L.B.-N.); hector.pelayo@correo.buap.mx (H.J.P.-G.); vicente.lopez@correo.buap.mx (V.A.L.-C.); maria.bonilla@correo.buap.mx (M.d.R.B.-S.); marco.garcia@correo.buap.mx (M.A.G.-F.); 2 Departamento de Ciencias Químico-Biológicas, Universidad de las Américas Puebla, San Andres Cholula 72810, Mexico; anac.hermosilloas@udlap.mx; 3 Instiute of Neurology, University College London (UCL), Queen Square, London WC1N 3BG, UK; diane.ruge@gmail.com; 4 Laboratoire de Recherche en Neurosciences Cliniques (LRENC), 34000 Montpellier, France; carlos.trenadoc@gmail.com; 5 Institute of Clinical Neuroscience and Medical Psychology, Heinrich Heine University, 40225 Dusseldorf, Germany; 6 Facultad de Ciencias de la Computación, Benemérita Universidad Autónoma de Puebla, Puebla 72000, Mexico; rafael.lemuz@correo.buap.mx

**Keywords:** ADHD, EEG, coherence, visual perception, school-aged children

## Abstract

Among neurodevelopmental disorders, attention deficit hyperactivity disorder (ADHD) is the main cause of school failure in children. Notably, visuospatial dysfunction has also been emphasized as a leading cause of low cognitive performance in children with ADHD. Consequently, the present study aimed to identify ADHD-related changes in electroencephalography (EEG) characteristics, associated with visual object processing in school-aged children. We performed Multichannel EEG recordings in 16-year-old children undergoing Navon’s visual object processing paradigm. We mapped global coherence during the processing of local and global visual stimuli that were consistent, inconsistent, or neutral. We found that Children with ADHD showed significant differences in global weighted coherence during the processing of local and global inconsistent visual stimuli and longer response times in comparison to the control group. Delta and theta EEG bands highlighted important features for classification in both groups. Thus, we advocate EEG coherence and low-frequency EEG spectral power as prospective markers of visual processing deficit in ADHD. Our results have implications for the development of diagnostic interventions in ADHD and provide a deeper understanding of the factors leading to low performance in school-aged children.

## 1. Introduction

### 1.1. ADHD in School-Aged Children

Children in elementary school not only face academic, social, and emotional challenges, but often also neurodevelopmental disorders, which lead to educational failure or abandonment. Attention deficit hyperactivity disorder (ADHD) is one such neurodevelopmental disorder [1] that has been extensively studied, and is also epidemiologically demonstrated to be a risk factor that determines shorter duration of education, poor long-term school outcomes, including high rates of school failure, and work disability in adulthood [2,3]. ADHD is considered a dysexecutive syndrome, where at least three psychophysiological mechanisms fail: (a) frontoparietal circuit, (b) frontostriatal circuit, and (c) frontotemporal circuit [4]. Neural circuitry integrity and organization are responsible for the appropriate functioning of the different cognitive functions that have a developmentally dynamic relationship with each other. Some studies have demonstrated cognitive comorbidities in children and adults with ADHD [5,6]. Children with ADHD show deficits in two neuropsychological mechanisms: spatial organization and behavioral modulation. Both mechanisms are fundamental for the acquisition of learning abilities [7]. 

The visuospatial functions arise from two neurofunctional brain systems, denominated ventral, and dorsal streams, differentiated by what (ventral stream) or how/where (dorsal stream) it is processed, but intricately related anatomically and functionally [8]. For that matter, visuospatial functioning has been implicated as one of the main abilities affected in children with ADHD [9]

It has been previously stated that visuospatial abilities exhibit different profiles among clinical (neurodevelopmental) groups, including ADHD. Most studies focus on the differences in visuospatial memory and the detrimental effect it can have on daily living, education, and working [10,11,12]. Research studies, focusing on local and global visual processing by means of Navon’s paradigm (hierarchical figures), have been directed to understand cross-disorder similarities/differences and to compare performance between children with and without ADHD [13,14,15].

However, some limitations of these studies are the age range of the population considered, which is not narrow enough to avoid developmental biases, and the fact that the authors often analyze visual processing in absence of other physiological measurements related to brain maturation/function. The interest in EEG maturation dates to the years following the publication of Hans Berger’s first article on EEG [16]; for example, the study of Lyndsay [17], about alpha rhythm in normal children. In this respect, Kellaway and Noebels [18] studied the normal alpha rhythm between the ages of 4 months to 16 years old, in which they reported that alpha rhythm in children of 11 years is about 9.7 Hz.

### 1.2. EEG Research Tools in Pediatric ADHD

Despite advances in our understanding of ADHD as a neuropsychological and neurodevelopmental disorder, robust biomarkers are yet to be established in clinical practice. After almost half a century, electroencephalography-based research has culminated in the recent Food and Drug Administration (FDA) approval of the theta/beta (EEG power) ratio (TBR) as a diagnostic marker of ADHD [19]. For that matter, some studies have approached markers of ADHD in children by using EEG measurements to gather information about ADHD brain-related electrical activity subtypes and functional connectivity. Nevertheless, such studies left aside the visuospatial function or considered it only a cognitive task, so a relationship between functional brain dynamics and visuospatial processing in ADHD has not yet been fully established [20,21,22,23,24].

Moreover, a recent systematic review study emphasized the importance of electrophysiological measures to provide meaningful insights into the heterogeneity of ADHD, although the direct translation of EEG biomarkers for diagnostic purposes is not yet supported. Key measures that show promise for the discrimination of existing ADHD subtypes and symptomatology include resting state and task-related modulation of alpha, beta, and theta power, and the event-related N2 and P3 components [25].

ADHD has been associated with atypical patterns of neural activity, measured by electroencephalography. However, the identification of EEG diagnostic biomarkers is complicated by the disorder’s heterogeneity [25]. Chen et al. [26], used evoked event-related potentials (ERP) during oddball P3 task. They found that healthy children recruited much stronger brain activity, mainly in the temporal and frontal regions, compared to ADHD children. Saad et al. [19], proposed that a personalized theta-to-alpha cut point or “transition frequency” is a better frame of reference for the measurement of Theta–Beta Ratio. Such an approach is better placed to test maturational lag and cortical hypo arousal models of ADHD and may, in turn, have greater utility in supporting diagnosis. Brier et al. [27], suggested that alpha rhythm over the posterior regions covary because of the effect of visual analytic function and the influence of theta rhythm over the frontal regions. The relations of theta–alpha may be considered as a type of functional system, related to the task condition. In other words, the relation of theta–alpha is induced for two possible reasons: (a) the discrimination of visual traits and (b) the control/inhibition for not failing the test.

Hager et al. [28], examined the associations between executive functions (EF), neuropsychological tests (including continuous performance), and ERP’s components in ADHD as a function of age. They found that age-group effects were seen on a selection of ERP amplitude. Ratings, test scores, and EF-related ERPs seem to capture different aspects of EF in ADHD, and the associations differed depending on age group. The results also showed that different measures of EF are not interchangeable and highlight the importance of age when interpreting ERPs. On the other hand, Hager et al. [29], argued that many studies applying EEG and neuropsychological tests found significant differences between ADHD and controls, but the effect sizes are often too small for diagnostic purposes. For this reason, they computed a diagnostic index for ADHD by combining behavioral test scores from a cued visual go/no-go task and event-related potentials (ERPs). They conclude that their diagnostic index has the potential to distinguish between ADHD and control groups.

Chen et al. [30], employed the power spectrum, complexity and bicoherence, biomarker candidates for identifying ADHD children in a machine learning approach, to characterize resting-state EEG (rsEEG). They conclude that the rsEEG complexity in ADHD children was significantly lower than controls and may be a suitable biomarker candidate. On the other hand, Furlong et al. [20], studying rsEEG connectivity in young children with ADHD, found that increased global efficiency (which measures the efficiency of information transfer across the entire brain) was associated with increased inattentive symptom severity. Further, this association was robust to controls for age, intelligent quotient, socioeconomic status, and internalizing psychopathology.

Smith et al. [31], examined the impact of an integrated brain, body, and social (IBBS) intervention (multi-faceted treatment consisting of computerized cognitive training, physical exercise, and behavior management) on ERPs of attentional control (P3 and N2) in children with ADHD. They conclude that prior to treatment, there was a significant difference between the ADHD group and the healthy control group for the N2 difference wave. Children with ADHD also showed slower reaction times on behavioral measures.

Lenartowicz and Loo [32], reviewed the developments in the utility of EEG in the diagnosis of ADHD, with emphasis on the most used and emerging EEG metrics and their reliability in diagnostic classification. They concluded that while EEG cannot currently be used as a diagnostic tool, vast developments in analytical and technological tools in its domain anticipate future progress in its utility in the clinical setting.

### 1.3. Age-Related Changes in EEG Coherence

Coherence changes can reflect the pathophysiological processes involved in human ageing. Barry et al. [33], studied the hemispheric and inter-hemispheric EEG coherences as a function of age and gender in normal children. They conclude that EEG coherences in normal children (8–12 years old) develop systematically with age. These developmental effects varied substantially with gender, brain region, and frequency bands. Furthermore, they investigated intra-hemispheric and inter-hemispheric EEG coherences as a function of age in boys [34] and girls [35], with different subtypes of ADHD, in comparison to a control group of normal boys and girls. They found that EEG coherences in normal boys of this age range develop systematically with age in a non-linear fashion. Boys with ADHD did not show this development. They displayed coherence anomalies, which differ in nature between DSM-IV subtypes, suggesting differences that are not relatable to simple symptom severity. However, girls with ADHD showed coherence anomalies relative to age- and gender-matched controls, which differ substantially from those shown by boys with ADHD. These coherence anomalies did not differ in nature between girls with different DSM-IV subtypes of ADHD, suggesting that subtype differences in girls reflect only symptom severity.

Thatcher et al. [36], explored the human development of EEG coherence and phase differences over the period from infancy to 16 years old. They found that large changes in EEG coherence and phase were present from 6 months to 4 years old, followed by a significant linear trend to higher coherence in short distance inter-electrode distances and longer phase delays in long inter-electrode distances. The results are consistent with a genetic model of rhythmic long-term connection formation that occurs in cycles along a curvilinear trajectory toward adulthood. Vysata et al. [37], studied a group of 17,722 healthy professional drivers and found a significant decrease in coherence with age in the theta and alpha bands, and there was an increasing coherence with the beta bands. The most prominent changes occurred in the alpha bands. The delta bands contained movement artefacts, which most likely do not change with age.

The aim of the study was to identify ADHD-related changes in EEG characteristics associated with visual object processing in school-aged children. Because there are very few studies describing EEG coherence during visual object recognition tests in children with ADHD, research in this area must be characterized, to understand their functional brain dynamics. In this respect, electroencephalographic coherence is a useful tool to assess connectivity and, therefore, functional brain dynamics [38,39,40], in combination with paradigms assessing the visual processing of objects, which represents a promising approach to study differences in brain function in children with and without ADHD. Because better neural connectivity affects brain functionality and, therefore, its behavioral performance, we hypothesize that the control group will have higher scores during visual aspect recognition and greater overall coherence during the Navon’s paradigm task than the experimental group.

## 2. Materials and Methods

### 2.1. Subjects

We implemented a cross-correlational study with non-probability statistical sampling for convenience. Sixteen school-aged children (11 years old) in the 5th grade at an urban elementary school in Puebla, Mexico, participated in this study. Age selection was in accordance with the period of psychological development and the guiding activity of the age range (7–11 years old). Namely, this period is when the main interest of children is directed toward school activity, and voluntary behavior and the capacity for theoretical individual learning of children represent desirable skills [41]. 

Experimental group consisted of 8 children diagnosed with ADHD by a child neurologist according to the following DSM-5 criteria: (1) lacks attention to detail, (2) difficulty sustaining attention, (3) fails to follow through on task and instruction, (4) poor organization, (5) avoids tasks requiring sustained mental effort, (6) loses things necessary for tasks, (7) easily distracted, (8) difficulties engaging in quiet, (9) talks excessively, (10) blurts out answers, (11) difficulty waiting turns, and (12) fidgets with or taps hands or feet. To double check the neurological diagnosis, we applied the neuropsychological assessment instrument “Child Neuropsychological Battery Puebla-Sevilla” to these children. The neuropsychological battery was designed to explore the integrity of the following neuropsychological mechanism: (1) arousal, (2) proprioceptive integrity, (3) serial movements, (4) visual and auditory memory, (5) inner images, (6) spatial organization, and (7) behavioral modulation. Control group consisted of 8 healthy children without ADHD. Children with any neurological condition, medicated and out of the established age range and school grade were excluded from this study. A Local Committee of the Faculty of Psychology approved the research protocol of this study on 12 January 2015. Experiments were conducted in accordance with the Declaration of Helsinki 1964. All children participated only after informed consent was obtained in written form from parents and orally from children. Socio-demographic variables for both groups are shown in Table 1. 

### 2.2. Navon Experimental Paradigm

#### 2.2.1. Global and Local Aspect Recognition 

**Figure 1:**
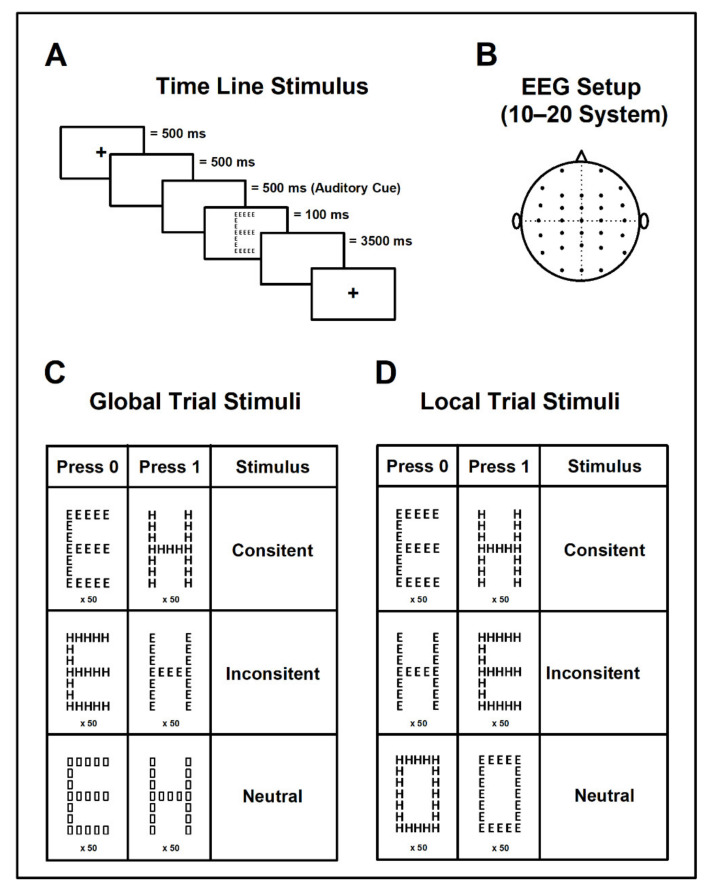
Hierarchical Visuospatial Aspect Recognition paradigm coupled with EEG. (**A**) The order and timing at which stimulus was presented were coupled with the EEG to create a window large enough for the collection of coherence data during visual object processing. (**B**) The placement of the electrodes on the scalp followed the international 10–20 system to assure standardization. (**C**) The stimuli selected for the Global Aspect Recognition trials comprised two consistent stimuli, two inconsistent and two neutral created with figures to avoid interference for the global visuospatial analysis. (**D**) The stimuli selected for the Local Aspect Recognition trials were the same for the consistent and inconsistent but differed from the global trial neutral stimuli since the global aspect created a figure (using the letters) to avoid interference of local visuospatial analysis.

To measure Global and Local (analytical) Aspect Recognition in schoolchildren, Navon’s hierarchical figures paradigm and the timeline stimulation was adapted and programmed in the Neuroscan STIM system (Compumedics, El Paso, TX, USA). Each trial of stimulation started with a 500 ms duration fixpoint. The screen then went blank for next 1000 ms. Subsequently, an auditory signal of 587 Hz was sounded for 500 ms. At the end of the auditory signal, the figure for visuospatial processing (global or local) was presented for 100 ms. The auditory cue ensured that the responses reflected only the visuospatial functioning and not a lack of attention. After this, the screen went blank and for 3500 ms the child was expected to respond. Therefore, the inter-trial intervals had a duration of 5100 ms. In each trial the first 1000 ms (just before the auditory cue) was considered as the EEG basal activity (Figure 1).

Visuospatial processing

1. Global Aspect Recognition Test: In this task children were asked to click on the response pad as soon as they identified the large figure (independently from the smaller figure composing it). We used 6 different types of stimuli: 2 consistent, 2 inconsistent, and 2 neutral. After the response, the entire trial began again by presenting a different stimulus (Figure 1A,C).
2. Local Aspect Recognition Test: In contrast to the first part, children were asked to identify the smaller figure without paying attention to the larger figure. The stimuli presented were also 2 consistent, 2 inconsistent, and 2 neutral; however, neutral stimuli were different for each test (global/local) (Figure 1D).

For both Local and Global Aspect Recognition Test, we presented each stimulus 50 times, so 600 stimuli were presented in total. The order in which target stimuli (consistent, inconsistent, neutral) appeared was randomized. Scores and latencies were stored for further analysis.

During the experimental session, the subject sat comfortably in an electrically shielded dimly lit room and the stimuli were presented on a 24″ monitor.

#### 2.2.2. Recordings

During the experimental session, children sat comfortably in an electrically shielded dimly lit room in front of a 24″ monitor that displayed the stimuli. We recorded EEG (band pass DC-200 Hz, sampling rate 1000 Hz) from 30 scalp positions referenced to earlobes with the ground at FzA, in accordance with the 10/20 system with the amplifier SynAmps, NeuroScan, (Compumedics, El Paso, TX, USA) (Figure 1B). Electrode impedances were kept under 5 kOhm. We also recorded the electrooculogram (same bandpass and sampling rate as for EEG) to exclude trials contaminated with eye movements for further analysis. Notch filter was set at 60 Hz. Behavioral data under Navon’s paradigm were recorded in parallel with the electrophysiological data. Data were stored and analyzed offline.

### 2.3. Data Analysis

#### 2.3.1. EEG-EEG Coherence Analysis

Data regarding EEG basal activity as well as Global and Local Aspect Recognition were included for analysis. We excluded segments contaminated with eye movement artifacts through visual inspection offline. EEG segments had a duration of 1000 ms, therefore, allowing a frequency resolution of 1 Hz for spectral analysis. We partitioned data into non-overlapping segments. The preprocess of EEG to quantify coherence was performed as in previous works [42,43,44]. EEG signals were first transformed into the reference-free current source density (CSD) distribution [45]. The CSD algorithm was estimated using the spherical spline interpolation method [46], implemented in the commercial software “Brain Vision 2.0.1” (Brain Vision Solutions Inc., München, Germany). For each subject, we had sequences of 80 segments of clean EEG (80 s). The discrete 2^9^ = 512-points Fourier transform was computed for each segment for the whole 0–200 Hz. Moreover, data belonging to global and local (consistent, inconsistent, and neutral) recognition from all trials were concatenated for further statistical analysis.

#### 2.3.2. Calculation of EEG Spectral Power and EEG-EEG Cortico-Cortical Coherence 

For the EEG Spectral Power (*SP*) and EEG-EEG Cortico-Cortical Coherence (CCC) calculation we apply the methodology used in previous works [42,43,44], which we briefly describe below. Because coherence function requires the complex values of *SP*, we first calculated *SP* for a given channel (*c*) according to the following formula:

(1) $$SP_{C}\left(f\right)=\frac{1}{n}\overset{n}{\underset{i=1}{\sum }}C_{i}\left(f\right)C_{i}^{*}\left(f\right)$$

where *C_i_* represents the Fourier transformed channel *c* for a given segment number (*i* = 1,…, *n*) and “*” indicates the complex conjugate. Then coherence values were calculated using the following formula:

(2) $$Coh_{C_{1}C_{2}}\left(f\right)=\frac{{\left|S_{C_{1}C_{2}}\left(f\right)\right|}^{2}}{\left|SP_{C_{1}}\left(f\right)\right|\left|SP_{C_{2}}\left(f\right)\right|}$$

where

(3) $$S_{C_{1}C_{2}}\left(f\right)=\frac{1}{n}\overset{n}{\underset{i=1}{\sum }}C_{1i}\left(f\right)C_{2i}^{*}\left(f\right)$$

Thus, *S_C_*_1,*C*2_(*f*) is the cross spectrum for the EEG signal channels *c*1 and *c*2 at a given frequency *f,* and *SP_C_*_1_(*f*) and *SP_C_*_2_(*f*) are the respective spectral power for *c*1 and *c*2 at the same frequency. The asterisk represents the complex conjugate. Thus, for frequency *f* the coherence value *Coh_C_*_1,*C*2_*(f)* corresponds to the squared magnitude of a complex correlation coefficient. The function $Coh_{C_{1}C_{2}}\left(f\right)\;$is a real number between 0 and 1, where 0 indicates absence of synchrony and 1 maximal synchrony between two signals. We considered that the coherence was significant if the resulting value isabove the confidence level (*CL*) [47].

(4) $$CL\left(\alpha \right)=1-{\left(1-\alpha \right)}^{\frac{1}{n-1}}$$

where the symbol *n* represents the number of segments of 512 points and the symbol α is the desired level of confidence. We considered that the coherence was significant when it was above the 95% confidence limit. For *n* = 80 segments and α = 0.95, *CL* = 0.037.

### 2.4. Calculation of Mean-Weighted Coherence

For the MWC we applied a modification methodology used in a previous work [48], which we briefly describe below. To quantify the EEG coherence, for a given pair of electrodes, we measured the area under the coherence curve and above the significance level. The frequency window was 1–50 Hz. 

We calculated all possible combinations (30 × 29/2 = 435) of coherence values between the EEG pair channels, which are the number of subsets or combinations without repetition of two elements taken from a set of 30. For a particular electrode we had 29 area values.

For each of the 30 electrodes, we averaged the sum of all 29 possible combinations of pairwise coherence for the electrode *i*. In this way we defined this number as the Mean-Weighted Coherence ($MWC_{i}$*_i_*) for the electrode *i*, which can be written as:

(5) $$MWC_{i}=\frac{\sum_{i=1}^{30}Coh\left(e_{i},e_{k}\right)}{29},$$

where *Coh*(*e*_i_,*e*_k_) is the EEG coherence between electrodes $e_{i}$, and $e_{k}$, and $WC_{i}$ is the Weighted Coherence for the electrode *i*,.

To obtain a general measure of the change in EEG synchronization, we defined the Global Mean-Weighted Coherence as:

(6) $$GMWC=\overset{30}{\underset{i=1}{\sum }}\overset{29}{\underset{k=1}{\sum }}Coh\left(e_{i},e_{k}\right)$$

To visualize the areas of the Mean-Weighted Coherence, we constructed a topographical map with each of the 30 values of *MWC*.

### 2.5. Statistical Analysis

We measured the Global Mean-Weighted Coherence for Global and Local Aspect Recognition in EEG basal activity (bEEG) as consistent (CC), inconsistent (IC), and neutral (NC) conditions. Because we wanted to know the contrast between control and ADHD group, we conducted statistical analysis on these two groups. Because our data were not normally distributed (Kolmogorov–Smirnov normality test, *p* < 0.05) and had no homogeneity of variances (Levene test, *p* < 0.05) we used a non-parametric U Mann–Whitney test, under the null hypothesis that the dependent variables were the same across the conditions. The statistical significance was calculated to one tail and all effects are reported as significant if *p* < 0.05.

## 3. Results

All sixteen subjects performed the task according to the instructions. None of them reported fatigue or anxiety during the experimental session. 

For intragroup, we used Friedman’s ANOVA for contrasts within groups and Wilcoxon test for post-hoc comparisons. Because we wanted to contrast the bEEG against the CC, IC, and NC, we applied Bonferroni correction. We report statistical significance below 0.0167.

We calculated EEG coherence by measuring the area under the curve and above the significance level. The frequency window was between 1 and 50 Hz. For each electrode, we calculated all 29 possible combinations of the MWC to obtain a general measure of change in synchronization between the different conditions for visual recognition.

**Figure 2:**
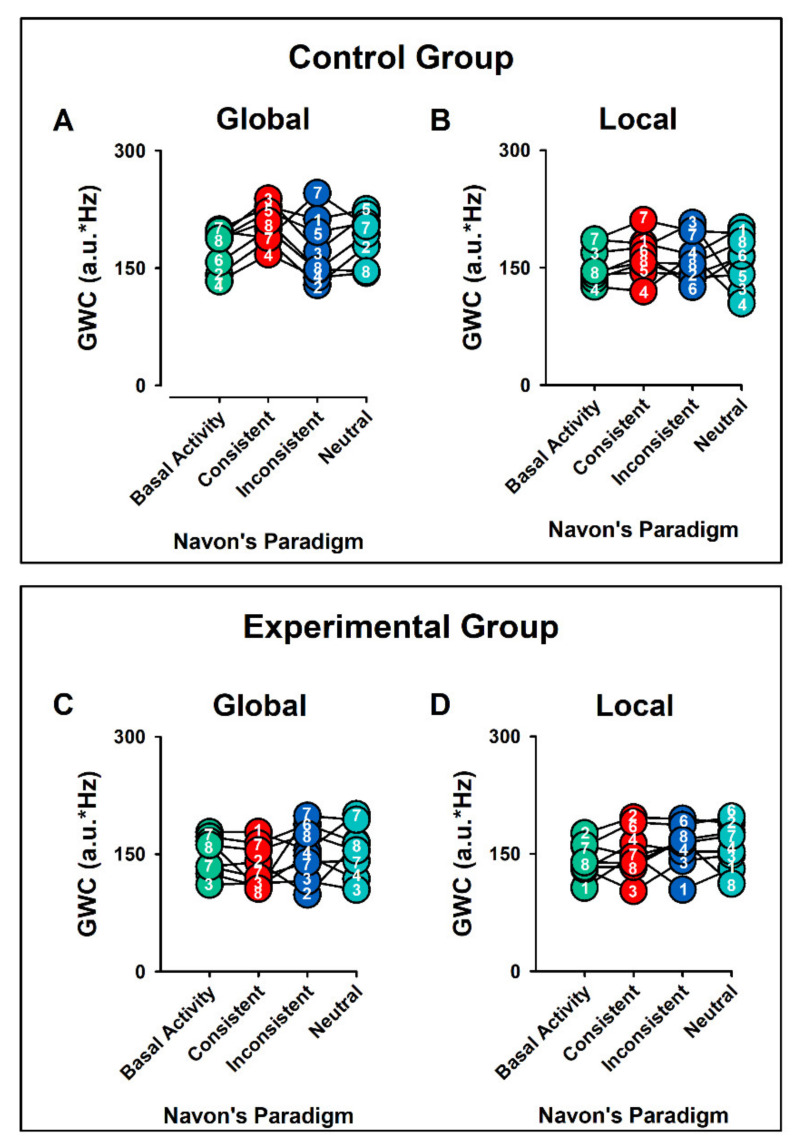
Pooled data for Global Mean-Weighted Coherence (GMWC), during Global (**A**,**C**) and Local (**B**,**D**) Aspect Recognition Test, for the control and experimental group, respectively. The individual values for each subject are represented with a number. In each case, the basal EEG activity, consistent, inconsistent, and neutral conditions are displayed. Note the interindividual differences. We observed higher values of GMWC in consistent and inconsistent Global Aspect Recognition in control group in comparison with ADHD group. Arbitrary units = a.u.; * = multiplication sign.

Figure 2 displays the individual values of GMWC, during Global (Figure 2A,C) and Local (Figure 2B,D) Aspect Recognition, for the control and experimental group, respectively. In each case, the bEEG, CC, IC, and NC are displayed.

### 3.1. Global Mean-Weighted Coherence

#### 3.1.1. Intergroup Contrast: Control vs. ADHD

##### Global Aspect Recognition

**Figure 3:**
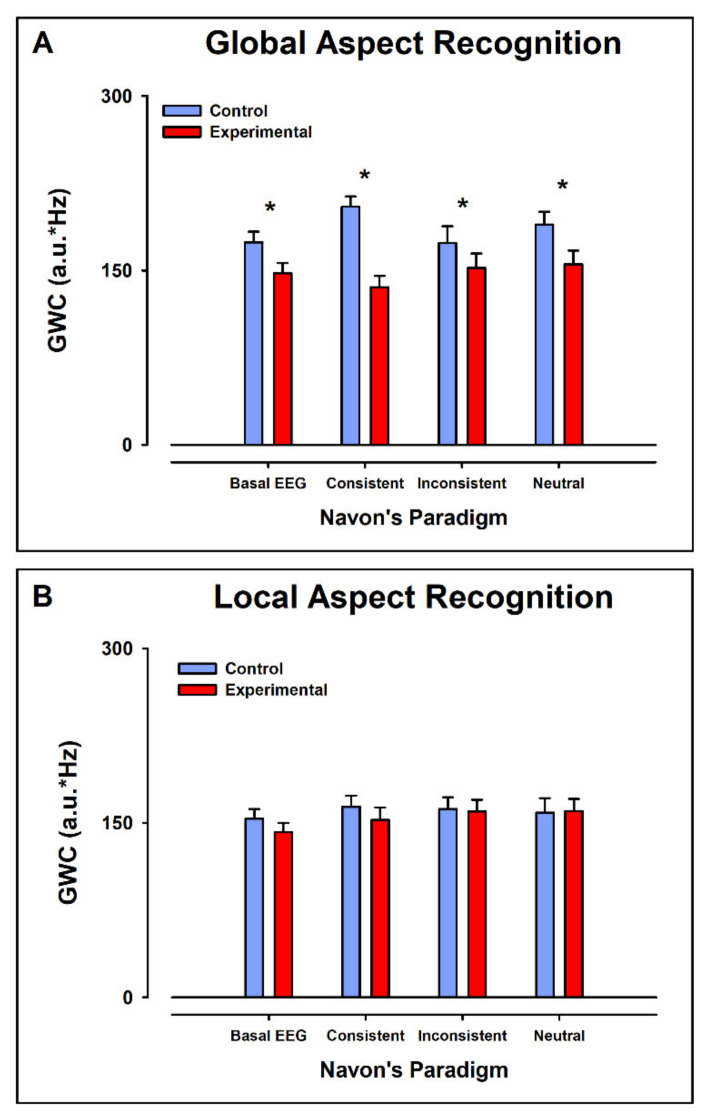
Comparison of the average Global Mean-Weighted Coherence (GMWC) obtained from control and experimental group. (**A**) GMWC for Global Aspect Recognition. (**B**) GMWC for Local Aspect Recognition. In (**A**,**B**), the basal EEG activity, consistent, inconsistent, and neutral conditions are displayed. Note that in A, there was a significant difference in the GMWC between the control group and the experimental group in all conditions. *p* < 0.05 is marked with *.

Figure 3 shows the means ± error of the data in Figure 2. For the Global Aspect Recognition test, the GMWC values were significantly larger in the control group than in the experimental group for the bEEG (Mdn_ctl_ = 174.2, Mdn_exp_ = 147.7, U = 14, z = −1.89 *p* < 0.05, r = −0.47), CC (Mdn_ctl_ = 173.5, Mdn_exp_ = 135.6, U = 15, z = −1.78 *p* < 0.05, r = −0.44), IC (Mdn_ctl_ = 205.0, Mdn_exp_ = 152.2, U = 6.0, z = −2.73 *p* < 0.05, r = −0.68) and NC (Mdn_ctl_ = 189.5, Mdn_exp_ =155.2, U = 14, z = −1.89 *p* < 0.05, r = −0.47) (Figure 3A). 

##### Local Aspect Recognition

However, for the Local Aspect Recognition test, the GMWC values in the control group did not differ significantly from the experimental group, in all conditions (bEEG, CC, IC, and NC) (Figure 3B).

#### 3.1.2. Intragroup Intra-Condition Contrasts

##### Control Group

In Global Aspect Recognition, Friedman’s ANOVA showed that the GMWC differed significantly in the bEEG, CC, IC, and NC, Chi^2^(3) = 10.95, *p* < 0.05. Wilcoxon tests were used to follow up this finding. A Bonferroni correction was applied and so all effects are reported at a 0.0167 level of significance. For the control group, the GMWC in CC (Mdn = 204.7) was significantly higher in comparison with the bEEG (Mdn =187.3), Z = −2.4, *p* < 0.0167, r = −0.59. However, we did not find significant differences in GMWC values between bEEG and IC, and bEEG and NC. 

In contrast, in Local Aspect Recognition, the GMWC values did not differ significantly in bEEG, CC, IC, and NC, Chi^2^(3) = 4.95, *p* > 0.05. 

##### Experimental Group

In Global Aspect Recognition, Friedman’s ANOVA showed that the GMWC values did not differ significantly in bEEG, CC, IC, and NC, Chi^2^(3) = 4.35, *p* > 0.05. 

Similarly, in Local Aspect Recognition, the GMWC values did not differ significantly in bEEG, CC, IC, and NC, Chi^2^(3) = 5.75, *p* > 0.05.

#### 3.1.3. Intragroup Contrasts Inter-Condition: Global vs. Local

For intragroup contrasts, between Global and Local Aspect Recognition, we used Friedman’s ANOVA with a Bonferroni correction of 0.016.

##### Control Group

The Friedman’s ANOVA showed that were significant differences between all conditions in global and local tasks, Chi^2^(8) = 24.1, *p* < 0.05. However, with the Bonferroni correction, we found that the post-hoc tests were not significantly different in all comparisons (global bEEG vs. local bEEG, global CC vs. local CC, global IC vs. local IC, and global NC vs. local IC). 

##### Experimental Group

Further, the Friedman’s ANOVA showed that there were no significant differences between all global and local conditions (bEEG, CC, IC, and NC), Chi^2^(8) = 10.7, *p* > 0.05. 

### 3.2. MWC Maps

**Figure 4:**
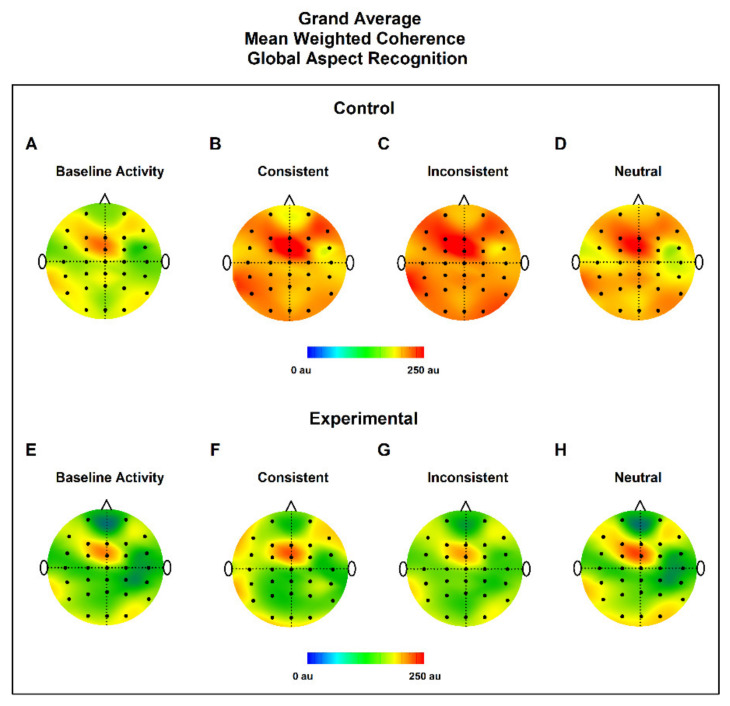
Grand average topographic maps of MWC for Global Aspect Recognition. Upper panel shows control group in EEG baseline activity (**A**), Consistent (**B**), Inconsistent (**C**) and Neutral conditions (**D**). Lower panel shows experimental group in EEG baseline activity (**E**), Consistent (**F**), Inconsistent (**G**) and neutral conditions (**H**). In all cases the maximum value of the MWC was found near FCZ located in the frontal lobe.

**Figure 5:**
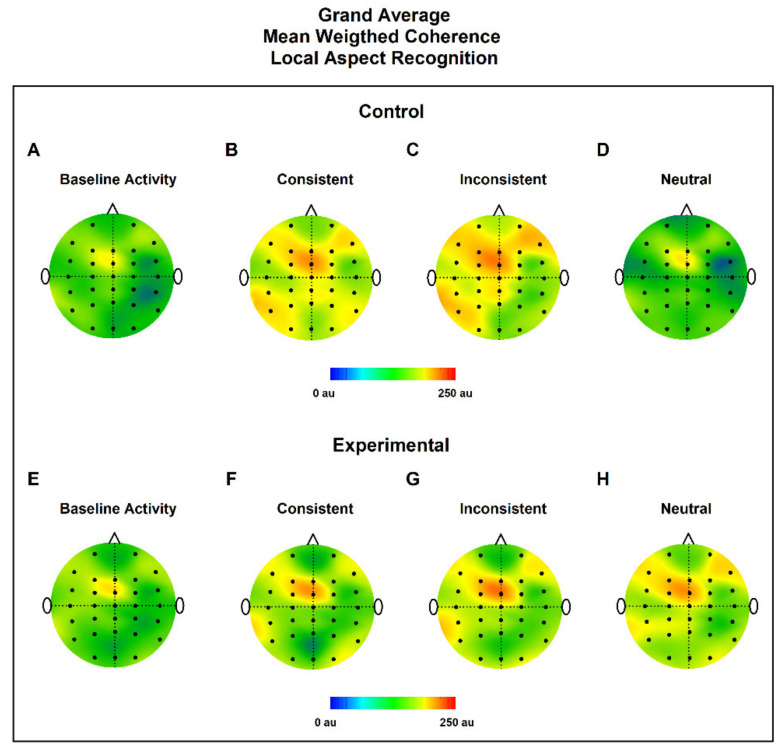
Grand average topographic maps of MWC for Local Aspect Recognition. Upper panel shows control group in EEG baseline activity (**A**), Consistent (**B**), Inconsistent (**C**) and Neutral conditions (**D**). Lower panel shows experimental group in EEG baseline activity (**E**), Consistent (**F**), Inconsistent (**G**) and neutral conditions (**H**). In all cases the maximum value of the MWC was found near FCZ located in the frontal lobe.

To have qualitative and quantitative information of coherence, we made grand average topographic maps of MWC for all subjects in all conditions (bEEG, CC, IC, and NC), for the control and experimental groups (Figure 4 and Figure 5). The control group showed the highest levels of MWC in all conditions in comparison to the experimental group. Qualitatively, we observed, in all conditions, that the highest values of MWC were in the frontal lobe, near FCZ.

### 3.3. Navon Scores 

**Figure 6:**
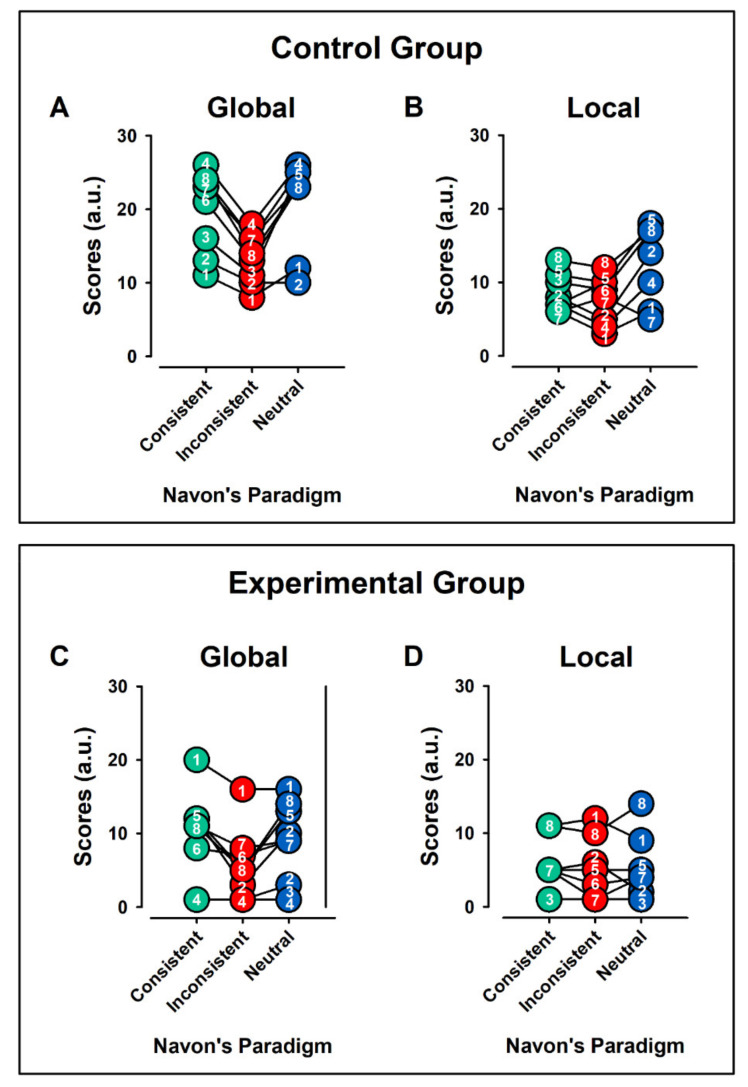
Pooled data of scores for all individuals, in Global (**A**,**C**) and Local (**B**,**D**) Aspect Recognition for control and experimental group, respectively. The individual values for each subject are represented with a number. In each case, the consistent, inconsistent, and neutral conditions are displayed. Note the interindividual differences. We observed higher values of scores in consistent Global Aspect Recognition in both groups.

**Figure 7:**
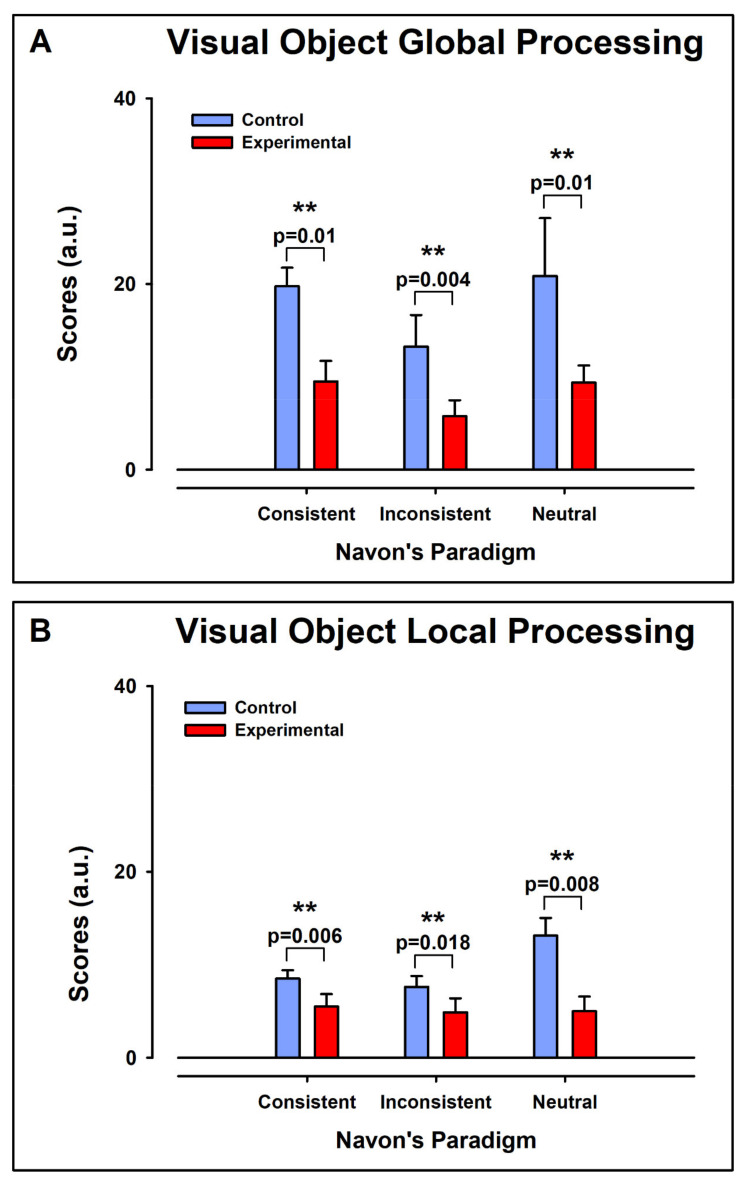
Comparison of the average scores of Navon test obtained from control and experimental group. (**A**) Scores for Global Aspect Recognition for consistent, inconsistent, and neutral stimulus. (**B**) Scores for Local Aspect Recognition for consistent, inconsistent, and neutral stimulus. Note that in all cases there was a significant decrease in the scores of the experimental group. *p* < 0.01 is marked with **.

Figure 6 displays the individual values of Navon Scores (NS) for the processing of Global (Figure 6A,C) and Local (Figure 6B,D) Aspect Recognition, for the control and experimental groups, respectively. In each case, the bEEG, CC, IC, and NC are displayed. Figure 7 shows the means ± error for the data in Figure 6.

#### 3.3.1. Intergroup Contrast: Control vs. ADHD

##### Global Aspect Recognition

For Global Aspect Recognition, the NS were significantly larger in the control group than in the experimental group for the CC (Mdn_ctl_ = 22.0 Mdn_exp_ = 11.0, U = 6.0, z = −2.75 *p* < 0.01, r = −0.68), IC (Mdn_ctl_ = 13.5, Mdn_exp_ = 5.0, U = 6.5, z = −2.69 *p* < 0.01, r = −0.67), and NC (Mdn_ctl_ = 23.0, Mdn_exp_ =9.5, U = 6.5, z = −2.70 *p* < 0.01, r = −0.68) (Figure 7A).

##### Local Aspect Recognition

For Global Aspect Recognition, the NS were significantly larger in the control group than in the experimental group for the CC (Mdn_ctl_ = 7.5, Mdn_exp_ = 5.0, U = 13.0, z = −2.02 *p* < 0.01, r = −0.68), and NC (Mdn_ctl_ = 15.5, Mdn_exp_ = 4.0, U = 6, z = −2.74 *p* < 0.01, r = −0.68). However, there were no differences in IC (Mdn_ctl_ = 8.5, Mdn_exp_ = 4.0, U = 19.5, z = −1.32 *p* > 0.05, r = −0.67) (Figure 7B).

#### 3.3.2. Intragroup Intra-Condition Contrasts

##### Control Group

In Global Aspect Recognition, Friedman’s ANOVA showed that the NS differed significantly in the CC, IC, and NC, Chi^2^(2) = 11.8, *p* < 0.05. Wilcoxon tests were used to follow up this finding. A Bonferroni correction was applied, so all effects are reported at a 0.025 level of significance. The values of NS in the NC were significantly larger (median = 23.0) than in the IC (median = 13.5), Z = −2.37, *p* < 0.025, r = 0.6 (Figure 7A). However, there were no differences between NC and CC.

On the other hand, in the Local Aspect Recognition, the NS differed significantly in the CC, IC and NC, Chi^2^(2) = 7.2, *p* < 0.05. Wilcoxon tests were used to follow up this finding. A Bonferroni correction was applied and so all effects are reported at a 0.025 level of significance. The values of NS in the NC were significantly larger (median = 15.0) than in the IC (median = 8.5), Z = −2.33, *p* < 0.025, r = 0.58 (Figure 7A). However, there were no differences between NC and CC.

##### Experimental Group

In Global Aspect Recognition, Friedman’s ANOVA showed the NS differed significantly in the CC, IC, and NC, Chi^2^(2) = 8.4, *p* < 0.05. Wilcoxon tests were used to follow up this finding. A Bonferroni correction was applied and so all effects are reported at a 0.025 level of significance. However, with the Bonferroni correction, we found that the post-hoc tests were not significantly different in all comparisons (NC vs. CC, and NC vs. IC).

#### 3.3.3. Intragroup Contrasts Inter-Condition: Global vs. Local

For control intragroup contrasts, between Global and Local Aspect Recognition, we used Friedman’s ANOVA for contrasts within groups, with a Bonferroni correction of 0.016.

##### Control Group

The Friedman’s ANOVA showed that there were significant differences in NS in global and local tasks in all conditions, Chi^2^(8) = 34.9, *p* < 0.05. Wilcoxon tests were used to follow up this finding. The values of NS in the global NC were significantly higher (median = 22.0) than in the local NC (median = 7.5), Z = −2.52, *p* < 0.025, r = 0.63 (Figure 7A). Further, NS in global IC (Median = 13.5) were significantly higher than in the local IC (median = 8.5), Z = −2.52, *p* < 0.025, r = 0.63 (Figure 7A). However, there were no differences between global CC vs. local CC.

##### Experimental Group

The Friedman’s ANOVA showed that there were significant differences in NS in global and local tasks in all conditions, Chi^2^(8) = 33.8, *p* < 0.05. Wilcoxon tests were used to follow up this finding. However, with the Bonferroni correction, we found that the post-hoc tests were not significantly different in all comparisons (global CC vs. local CC, global IC vs. local IC, and global NC vs. local IC).

### 3.4. Latencies

**Figure 8:**
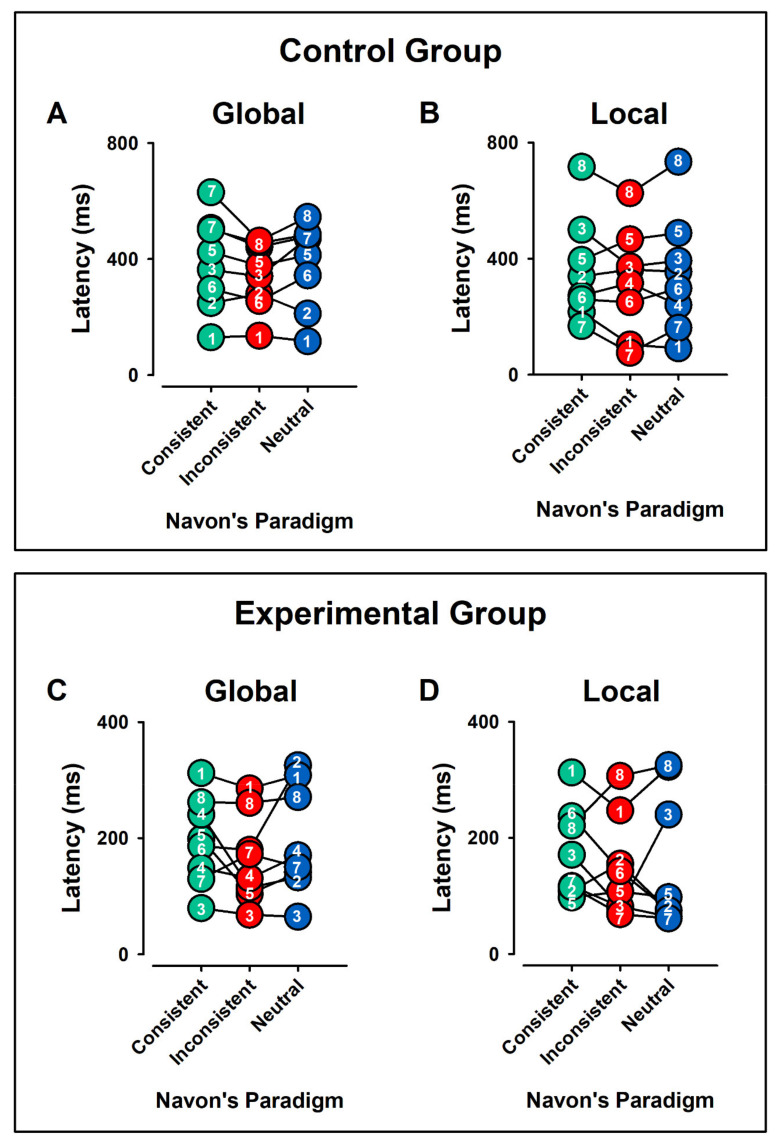
Pooled data of latencies for all individuals in Global (**A**,**C**) and Local (**B**,**D**) Aspect Recognition for control and experimental groups, respectively. The individual values for each subject are represented with a number. In each case, the consistent, inconsistent, and neutral conditions are displayed. Note the interindividual differences. We observed higher latencies in control group than in experimental group.

Figure 8 displays the individual values of latencies for the processing of global (Figure 8A,C) and local (Figure 8B,D) visual stimuli, for the control and experimental groups, respectively. In each case, the consistent, inconsistent, and NCs are displayed. 

**Figure 9:**
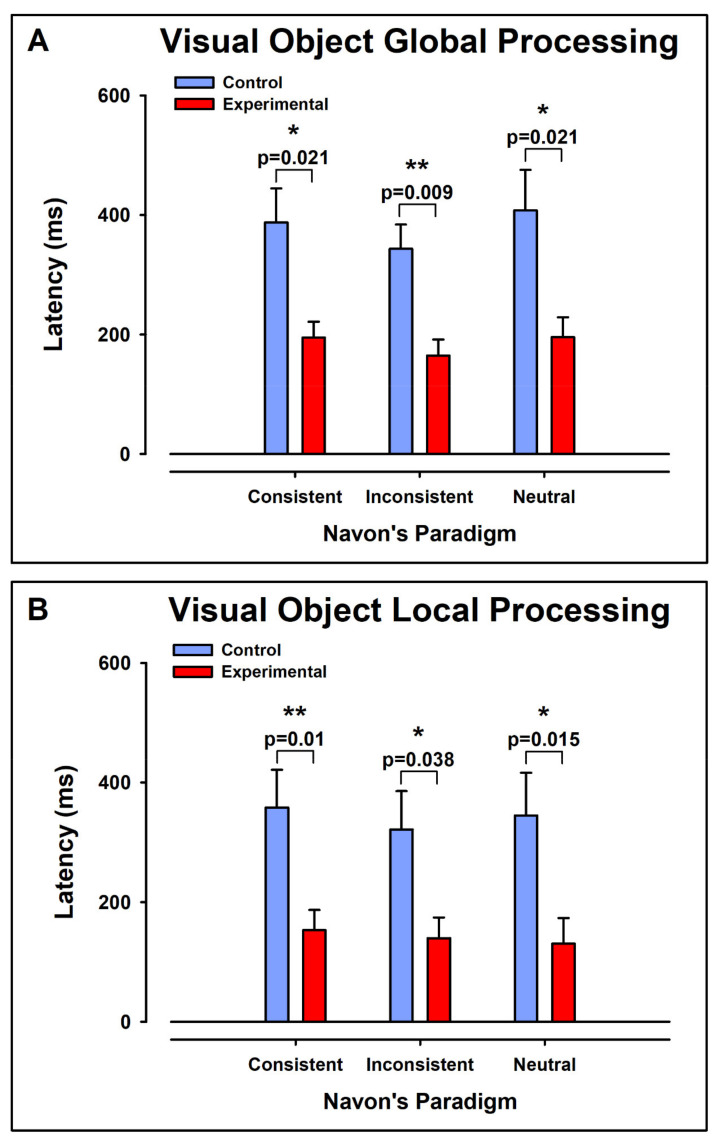
Comparison of the average latencies obtained from control and experimental groups. (**A**) Latencies for Global Aspect Recognition for consistent, inconsistent, and neutral stimulus. (**B**) Latencies for Local Aspect Recognition for consistent, inconsistent, and neutral stimulus. Note that in all cases there was a significant decrease in the latencies of the experimental group. *p* < 0.05 is marked with * and *p* < 0.01 is marked with **.

Figure 9 shows the means ± error for the data in Figure 8.

#### 3.4.1. Intergroup Contrast: Control vs. ADHD

##### Global Aspect Recognition

For the Global Aspect Recognition, the latencies were significantly larger in the control group than in the experimental group for the CC (Mdn_ctl_ = 393.5 ms, Mdn_exp_ = 192.4 ms, U = 10, z = −2.31 *p* < 0.05, r = −0.57), IC (Mdn_ctl_ = 358.9 ms, Mdn_exp_ = 151.6 ms, U = 7.0, z = −2.63 *p* < 0.01, r = −0.66), and NC (Mdn_ctl_ = 442.7 ms, Mdn_exp_ =160.3, U = 10.0, z = −2.31 *p* < 0.05, r = −0.57) (Figure 9A).

##### Local Aspect Recognition

For the local visual processing, the latencies were significantly larger in the control group than in the experimental group for the CC (Mdn_ctl_ = 305.8 ms, Mdn_exp_ = 116.0 ms, U = 8.0, z = −2.52 *p* < 0.01, r = −0.63), IC (Mdn_ctl_ = 338.8 ms, Mdn_exp_ = 125.1 ms, U = 12, z = −2.10 *p* < 0.05, r = −0.52), and NC (Mdn_ctl_ = 326.0 ms, Mdn_exp_ = 74.8 ms, U = 9, z = −2.41 *p* < 0.05, r = −0.60). 

#### 3.4.2. Intragroup Intra-Condition Contrasts

##### Control Group

In Global Aspect Recognition, Friedman’s ANOVA showed that latencies did not differ significantly in the CC, IC, and NC, Chi^2^(2) = 3.25, *p* > 0.05. 

On the other hand, in Local Aspect Recognition, latencies did not differ significantly in the CC, IC, and NC, Chi^2^(2) = 0.75, *p* > 0.05.

##### Experimental Group

In Global Aspect Recognition, Friedman’s ANOVA showed that latencies did not differ significantly in the CC, IC, and NC, Chi^2^(2) = 4.75, *p* > 0.05. 

On the other hand, in Local Aspect Recognition the latencies did not differ significantly in the CC, IC, and NC, Chi^2^(2) = 0.25, *p* > 0.05. 

#### 3.4.3. Intragroup Contrasts Inter-Condition: Global vs. Local Condition 

##### Control and Experimental Group

Friedman’s ANOVA showed that in control group, latencies did not differ significantly in the CC, IC, and NC in Global and Local Aspect Recognition, Chi^2^(2) = 4.43, *p* > 0.05. Further, in the experimental group, latencies did not differ significantly in the CC, IC, and NC in Global and Local Aspect Recognition, Chi^2^(2) = 10.8, *p* > 0.05.

## 4. Discussion

We focused on studying coherence at a wide bandwidth (1–50 Hz), with the purpose of having a general indicator for global-weighted coherence, leaving, for future studies, a more local analysis in certain frequency bands. We analyzed up to 50 Hz because the origin of frequencies beyond this value is not considered to be of physiological origin.

Coherence is a dimensionless measure of synchrony between two signals (labeled in the text as arbitrary units = a.u.), while other technics, such as ERPs, are time-averaged events and only give an indicator of neuronal ensemble activation. Other technics to analyze EEG include Spectral Power, which provides an indicator for the energy captured by the electrode at a certain frequency.

We analyzed EEG in this study with Cortico-Cortical Coherence. However, a disadvantage of EEG coherence is that the recording electrodes are not in direct contact with the nerve or neuronal assemblies and, therefore, there is a medium of some sort separating the two sites of recording. This is why, to reduce the volume conductor, EEG signals were first transformed into the reference-free current source density (CSD) distribution [45]. On the other hand, one advantage of coherence is that it allows us to have an indicator for the degree of synchrony in the electrical activity of the neuronal assemblies underlying the registration site. This synchrony is interpreted physiologically and functionally as neuronal connectivity. Our goal, in this study, was to give a global perspective about this functional connectivity, in the context of Navon’s paradigm.

In this work, we were able to identify changes in coherence from electroencephalographic signals in a sample of school-aged children diagnosed with ADHD, associated with visual aspect recognition. We found a statistically significant lower global coherence in children with ADHD in comparison to healthy children, during Global Aspect Recognition in the consistent, inconsistent, and neutral conditions. Furthermore, in the ADHD group, we found that their scores were characterized by lower efficiency and lower latencies for all stimuli, in comparison with control. A possible explanation is that lower latencies are due to the impulsivity of the children and possibly that they were not medicated. This in line with Sayal et al. [49], who reported that patients with ADHD are characterized by being impulsive. In contrast with our results, Smith et al. [31], reported slower reaction times on behavioral measures in children with ADHD. 

ADHD errors can be caused by the lack of an organized and precise perception for the dominant characteristics of an object. Luria [50] stated that the perception of visual information is an active process of selection of both essential and differential meanings of visual objects, in which the comparison of perceived details (i.e., the formation of a hypothesis about the meaning of the whole) and the choice of them, give a general meaning of the complete image, from several possible alternatives.

Likewise, the low performance obtained by the children with ADHD may be due to little active object exploration. It was observed that participants with ADHD did not carry out a systematic process of visual analysis of the determining details in the image, they did not compare or submit the details of their analysis, and their last hypothesis did not arise from a complex activity of investigative orientation, for which reason, the responses emitted are the product of an immediate impression (impulsive) by the perception of a single fragment of the image.

This in line with previously reported studies in the neuropsychological literature of patients with ADHD, who are characterized by being impulsive, excitable, lacking self-control and reflecting on their own disorganized behavior [49].

Although the sample size was small, still, implications of specific results can be drawn, as follows. Global Aspect Recognition in the control group induced greater coherence in the frontal medial and central leads, mainly in the consistent and inconsistent condition. This suggests that concentrated (focal) activation in these areas is necessary for global processing. These areas seem to organize a kind of open and dynamic exploration. On the other hand, visual stimuli of a local nature in the control group, under the consistent and inconsistent experimental conditions, induced greater coherence in the frontal medial and central leads but also over the polar frontal, inferior frontal and left temporal leads in both groups. This activation requires a greater effort and expenditure of neuronal resources. One possible interpretation could be that visual scanning (medial frontal) attempts to identify stimuli that are familiar or have been previously recorded (medial temporal areas). 

In contrast, for the ADHD group, global visual stimuli produced greater coherence in the medial frontal. The analysis of visual stimuli of a local nature in this group generated greater coherence in the medial frontal, inferior frontal, and polar frontal leads, but less coherence compared with the control group over the left temporal leads, predominantly in the left hemisphere.

The results suggest that visual processing is not an isolated function and that it also requires motor control and rapid stimuli encoding. This evidence seems to suggest that tasks that require greater cognitive effort per attention induce greater coherence in brain systems that are evolutionarily essential to meet these demands.

Abnormal functional brain connectivity is a candidate factor in brain development disorders associated with cognitive dysfunction. Murias et al. [22], compared the EEG of children with ADHD with a healthy group. In response to visual stimulation, the ADHD group exhibited an increase in spectral power and an increase in elevated frontal coherence. Only the increase in the coherence of the control group in the upper alpha band was discriminated according to the degree of medication in the ADHD group. The results suggested a static state of poor connectivity in ADHD and a state of stimulus-induced hyperconnectivity, within and between frontal hemispheres. Moreover, Sehatpour et al. [51], found coherence in the beta band during the processing of fragmented recognizable images. This coherence was significantly lower compared to visual processing of the same figure but unrecognizably fragmented. Long-range wobble synchronization has been proposed as a better way to coordinate a distributed network. It is likely that the low levels of coherence we found in the ADHD group during visual recognition, in comparison with the control group, are due to the mechanisms proposed by Murias et al. [22], and Sehatpour et al. [51].

Other experimental paradigms have been used to study the neural basis of visual processing in different contexts. For example, Brummerloh et al. [52], used attention based on visual processing of the characteristics (rotation and color) of an object (square) to study temporal neural dynamics. Their study concluded that timing is important, as brief stimulus presentations, in the range of a few hundred milliseconds, resulted in the integration of object features. However, when the stimulation time was longer, attentional mechanisms based on the characteristics of the object predominated, resulting in the specific prioritization of the characteristic to be attended to within an object. This is in line with our results, since the stimulation time in our paradigm was short (100 ms) and the subjects in the control group correctly integrated the characteristics of the figures.

Coherent neural oscillations correlate with all cognitive functions, mediating local and long-range neural communication and affecting synaptic plasticity. Although we know that the EEG changes with age (and, therefore, all the observables associated with it, such as Spectral Power, coherence, wavelets, LORETA, etc.), EEG behavior depends on the context of the investigation. In our research, age was homogeneous for all participants (11 years old), so the differences in brain maturation would not affect our results interpretations. However, it is unclear how very rapid and complex changes in functional neuronal connectivity are required for cognition, mediated by dynamic patterns of neuronal synchrony, which could be explained exclusively by well-established synaptic mechanisms [53]. 

In our study, we found the highest levels of global-weighted coherence in the control group during the Global Aspect Recognition. Furthermore, in this group, we found the highest scores from the Navon test. However, we did not find any correlation between them. Several studies suggest that both task-relevant and task-irrelevant features of an object will be processed together without specific prioritization [54,55].

Rizkallah et.al. [56], questioned whether the dynamic brain modular organization changes during the recognition of meaningful and nonsense visual images. Their results showed a difference in the characteristics in the modular organization of both conditions, in terms of integration (interactions between modules) and occurrence (average probability of two brain regions to fall on the same module during the task). Integration and occurrence were higher for images without meaning than with meaning. Their findings also revealed that occurrence within the right frontal and left occipital-temporal regions can help predict the brain’s ability to recognize and name visual stimuli. They speculate that these observations could be applicable not only to other fast-demanding cognitive functions but also to detect rapid disconnections that can occur in some brain disorders.

Gorantla et al., [57], conducted a study in 10 medical students, in which they measured the EEG three days before an anatomy exam. This research found that decreased theta–beta ratio (TBR) is an indicator of attentional control and was associated with higher alpha and beta interhemispheric coherences, measured with eyes open at sites covering the frontal, temporal, and occipital cortices. These authors concluded that changes in EEG and TBR coherence may be useful as neurophysiological measures of neuroplasticity and the efficacy of strategies to prevent academic underachievement and to improve academic achievement strategies.

### 4.1. Limitations

One limitation of our study resides in the size of the population included. We expect that, when broadened in future studies, differences in the coherence patterns observed so far would be observed. In this way valuable information regarding specific patterns related to ADHD could be obtained in future research if the examination of data is broadened in such a direction. 

### 4.2. Open Questions for Further Research

Overall, more research along these lines is needed, especially with larger samples, but the results obtained so far imply that it is possible to delineate an ADHD-specific pattern of coherence that will aid in clinical diagnosis and allow for the creation of tailored rehabilitation and educational intervention programs, based on the neurobiology and neuropsychology of the disorder. We also believe that future work should include a more specific analysis in the Delta (0.5–4 Hz), Theta (4–8 Hz), Alpha (8–12 Hz), Beta (12–30 Hz), and Gamma (30–45 Hz) bands. Therefore, specific MWC estimates for these frequency bands could discover more detail on the observed effects. Moreover, further analysis should include wavelet analyses to extract quantitative indicators from EEG segments to classify the characteristics of the child population with ADHD, in a similar way to how Aidyn et al. [58], classified the emotional characteristics of patients diagnosed with first-episode psychosis, using Cortical Correlations in the wavelet domain.

## 5. Conclusions

Using the proposed algorithm, we showed that, in principle, it is possible to recognize if every single task of Global Aspect Recognition in Navon’s experiment has been solved by a mentally healthy child or a child diagnosed with ADHD. We conclude that while our promising classification results obtained using a small EEG database cannot currently be used as a diagnostic tool, the information encoded in the signals is highly discriminative and we plan to extend the study, analyzing information in a larger population, towards the goal of developing a tool that can be used in educational and clinical settings. Specifically, given the problematic situation of ADHD sufferers in the school system, technical approaches, such as the one presented here, with EEG-based data, could pave the way towards the identification of risk for a student and development towards an individualized teaching approach. 

## Figures and Tables

**Table 1:** Socio-demographic variables.

Group	Mean Age (SD)	School	Scholarship	Female	Male	Current Drug Use
Control	11 (0)	Elementary Urban Public	5th grade	5	3	NO
ADHD				4	4	NO

## Data Availability

The datasets generated for this study are available on request to the corresponding author.

## References

[1] Scandurra V., Emberti Gialloreti L., Barbanera F., Scordo M.R., Pierini A., Canitano R. (2019). Neurodevelopmental Disorders and Adaptive Functions: A Study of Children with Autism Spectrum Disorders (ASD) and/or Attention Deficit and Hyperactivity Disorder (ADHD). Front. Psychiatry.

[2] Barbaresi W.J., Katusic S.K., Colligan R.C., Weaver A.L., Jacobsen S.J. (2007). Long-Term School Outcomes for Children with Attention-Deficit/Hyperactivity Disorder: A Population-Based Perspective. J. Dev. Behav. Pediatr..

[3] Fredriksen M., Dahl A.A., Martinsen E.W., Klungsoyr O., Faraone S.V., Peleikis D.E. (2014). Childhood and persistent ADHD symptoms associated with educational failure and long-term occupational disability in adult ADHD. ADHD Atten. Deficit Hyperact. Disord..

[4] Diamond A. (2005). Attention-Deficit Disorder (Attention-Deficit/Hyperactivity Disorder without Hyperactivity): A Neurobiologically and Behaviorally Distinct Disorder from Attention-Deficit/Hyperactivity Disorder (with Hyperactivity). Dev. Psychopathol..

[5] Tirosh E., Cohen A. (1998). Language deficit with attention-deficit disorder: A prevalent comorbidity. J. Child Neurol..

[6] Marks D.J., Newcorn J.H., Halperin J.M. (2001). Comorbidity in adults with attention-deficit/hyperactivity disorder. Ann. N. Y. Acad. Sci..

[7] Quintanar L., Solovieva Y., Bonilla R. (2006). Analysis of Visuospatial Activity in PreschoolChildren with Attention Deficit Disorder. Hum. Physiol..

[8] Stiles J., Akshoomoff N.A., Haist F. (2020). The development of visuospatial processing. Neural Circuit and Cognitive Development.

[9] Soluki S., Nejati V., Fathabadi J. (2020). Spatial Ability in children with Attention-Deficit/Hyperactivity Disorder (ADHD) and its Impact on Executive Functions. Res. Sq..

[10] Alderson R.M., Kasper L.J., Hudec K.L., Patros C.H.G. (2013). Attention-deficit/hyperactivity disorder (ADHD) and working memory in adults: A meta-analytic review. Neuropsychology.

[11] Kofler M.J., Sarver D.E., Spiegel J.A., Day T.N., Harmon S.L., Wells E.L. (2017). Heterogeneity in ADHD: Neurocognitive Predictors of Peer, Family, and Academic Functioning. Child Neuropsychol..

[12] Rapport M.D., Orban S.A., Kofler M.J., Friedman L.M. (2013). Do programs designed to train working memory, other executive functions, and attention benefit children with ADHD? A meta-analytic review of cognitive, academic, and behavioral outcomes. Clin. Psychol. Rev..

[13] Cardillo R., Vio C., Mammarella I.C. (2020). A comparison of local-global visuospatial processing in autism spectrum disorder, nonverbal learning disability, ADHD and typical development. Res. Dev. Disabil..

[14] Ging-Jehli N.R., Ratcliff R., Arnold L.E. (2021). Improving neurocognitive testing using computational psychiatry—A systematic review for ADHD. Psychol. Bull..

[15] Song Y., Hakoda Y. (2015). Lack of global precedence and global-to-local interference without local processing deficit: A robust finding in children with attention-deficit/hyperactivity disorder under different visual angles of the Navon task. Neuropsychology.

[16] Berger H. (1934). Über das elektrenkephalogramm des menschen. DMW-Dtsch. Med. Wochenschr..

[17] Lindsley D.B. (1939). A longitudinal study of the occipital alpha rhythm in normal children: Frequency and amplitude standards. Pedagog. Semin. J. Genet. Psychol..

[18] Kellaway P., Noebels J.L. (1989). Problems and Concepts in Developmental Neurophysiology.

[19] Saad J.F., Kohn M.R., Clarke S., Lagopoulos J., Hermens D.F. (2018). Is the theta/beta EEG marker for ADHD inherently flawed?. J. Atten. Disord..

[20] Furlong S., Cohen J.R., Hopfinger J., Snyder J., Robertson M.M., Sheridan M.A. (2020). Resting-state EEG Connectivity in Young Children with ADHD. J. Clin. Child Adolesc. Psychol..

[21] Khoshnoud S., Nazari M.A., Shamsi M. (2018). Functional brain dynamic analysis of ADHD and control children using nonlinear dynamical features of EEG signals. J. Integr. Neurosci..

[22] Murias M., Swanson J.M., Srinivasan R. (2007). Functional Connectivity of Frontal Cortex in Healthy and ADHD Children Reflected in EEG Coherence. Cereb. Cortex.

[23] Robbie J.C., Clarke A.R., Barry R.J., Dupuy F.E., McCarthy R., Selikowitz M. (2016). Coherence in children with AD/HD and excess alpha power in their EEG. Clin. Neurophysiol..

[24] Zarafshan H., Khaleghi A., Mohammadi M.R., Moeini M., Malmir N. (2016). Electroencephalogram complexity analysis in children with attention-deficit/hyperactivity disorder during a visual cognitive task. J. Clin. Exp. Neuropsychol..

[25] Slater J., Joober R., Koborsy B.L., Mitchell S., Sahlas E., Palmer C. (2022). Can electroencephalography (EEG) identify ADHD subtypes? A systematic review. medRxiv.

[26] Chen C., Yang H., Du Y., Zhai G., Xiong H., Yao D., Xu P., Gong J., Yin G., Li F. (2021). Altered Functional Connectivity in Children with ADHD Revealed by Scalp EEG: An ERP Study. Neural Plast..

[27] Brier M.R., Ferree T.C., Maguire M.J., Moore P., Spence J., Tillman G.D., Tillman J.H., Kraut M.A. (2010). Frontal theta and alpha power and coherence changes are modulated by semantic complexity in Go/NoGo tasks. Int. J. Psychophysiol..

[28] Häger L.A., Øgrim G., Danielsen M., Billstedt E., Gillberg C., Johnels J.Å. (2020). Indexing Executive Functions with Test Scores, Parent Ratings and ERPs: How Do the Measures Relate in Children versus Adolescents with ADHD?. Neuropsychiatr. Dis. Treat..

[29] Häger L.A., Johnels J.Å., Kropotov J.D., Weidle B., Hollup S., Zehentbauer P.G., Gillberg C., Billstedt E., Ogrim G. (2021). Biomarker support for ADHD diagnosis based on Event Related Potentials and scores from an attention test. Psychiatry Res..

[30] Chen H., Chen W., Song Y., Sun L., Li X. (2019). EEG characteristics of children with attention-deficit/hyperactivity disorder. Neuroscience.

[31] Smith S.D., Crowley M.J., Ferrey A., Ramsey K., Wexler B.E., Leckman J.F., Sukhodolsky D.G. (2019). Effects of Integrated Brain, Body, and Social (IBBS) intervention on ERP measures of attentional control in children with ADHD. Psychiatry Res..

[32] Lenartowicz A., Loo S.K. (2014). Use of EEG to diagnose ADHD. Curr. Psychiatry Rep..

[33] Barry R.J., Clarke A.R., McCarthy R., Selikowitz M., Johnstone S.J., Rushby J.A. (2004). Age and gender effects in EEG coherence: I. Developmental trends in normal children. Clin. Neurophysiol..

[34] Barry R.J., Clarke A.R., McCarthy R., Selikowitz M. (2005). Age and gender effects in EEG coherence: II. Boys with attention-deficit/hyperactivity disorder. Clin. Neurophysiol..

[35] Barry R.J., Clarke A.R., McCarthy R., Selikowitz M. (2006). Age and gender effects in EEG coherence: III. Girls with attention-deficit/hyperactivity disorder. Clin. Neurophysiol..

[36] Thatcher R.W., North D.M., Biver C.J. (2008). Development of cortical connections as measured by EEG coherence and phase delays. Hum. Brain Mapp..

[37] Vysata O., Kukal J., Prochazka A., Pazdera L., Simko J., Valis M. (2014). Age-related changes in EEG coherence. Neurol. I Neurochir. Pol..

[38] Babiloni C., Blinowska K., Bonanni L., Cichocki A., De Haan W., Del Percio C., Dubois B., Escudero J., Fernández A., Frisoni G. (2020). What electrophysiology tells us about Alzheimer’s disease: A window into the synchronization and connectivity of brain neurons. Neurobiol. Aging.

[39] Mehdizadefar V., Ghassemi F., Fallah A. (2019). Brain Connectivity Reflected in Electroencephalogram Coherence in Individuals with Autism: A Meta-Analysis. Basic Clin. Neurosci..

[40] Miraglia F., Vecchio F., Rossini P.M. (2018). Brain electroencephalographic segregation as a biomarker of learning. Neural Netw..

[41] El’Konin D.B. (1971). Toward the problem of stages in the mental development of children. Vopr. Psikhologii.

[42] Trenado C., Mendez-Balbuena I., Manjarrez E., Huethe F., Schulte-Mönting J., Feige B., Hepp-Reymond M.C., Kristeva R. (2014). Enhanced corticomuscular coherence by external stochastic noise. Front. Hum. Neurosci..

[43] Mendez-Balbuena I., Huethe F., Schulte-Mönting J., Leonhart R., Manjarrez E., Kristeva R. (2012). Corticomuscular coherence reflects interindividual differences in the state of the corticomuscular network during low-level static and dynamic forces. Cereb. Cortex.

[44] Omlor W., Patino L., Mendez-Balbuena I., Schulte-Mönting J., Kristeva R. (2011). Corticospinal beta-range coherence is highly dependent on the pre-stationary motor state. J. Neurosci..

[45] Nunez P.L., Srinivasan R., Westdorp A.F., Wijesinghe R.S., Tucker D.M., Silberstein R.B., Cadusch P.J. (1997). EEG coherency. I: Statistics, reference electrode, volume conduction, Laplacians, cortical imaging, and interpretation at multiple scales. Electroencephalogr. Clin. Neurophysiol..

[46] Perrin F., Pernier J., Bertrand O., Echallier J.F. (1989). Spherical splines for scalp potential and current density mapping. Electroencephalogr. Clin. Neurophysiol..

[47] Rosenberg J.R., Amjad A.M., Breeze P., Brillinger D.R., Halliday D.M. (1989). The Fourier approach to the identification of functional coupling between neuronal spike trains. Prog. Biophys. Mol. Biol..

[48] Mendez-Balbuena I., Arrieta P., Huidobro N., Flores A., Lemuz-Lopez R., Trenado C., Manjarrez E. (2018). Augmenting EEG-global-coherence with auditory and visual noise: Multisensory internal stochastic resonance. Medicine.

[49] Sayal K., Prasad V., Daley D., Ford T., Coghill D. (2018). ADHD in children and young people: Prevalence, care pathways, and service provision. Lancet Psychiatry.

[50] Luria A.R. (2003). The role of language in the formation of temporary connections. Psychology in the Soviet Union Ils 272.

[51] Sehatpour P., Molholm S., Schwartz T.H., Mahoney J.R., Mehta A.D., Javitt D.C., Stanton P.K., Foxe J.J. (2008). A human intracranial study of long-range oscillatory coherence across a frontal-occipital-hippocampal brain network during visual object processing. Proc. Natl. Acad. Sci. USA.

[52] Brummerloh B., Müller M.M. (2019). Time matters: Feature-specific prioritization follows feature integration in visual object processing. NeuroImage.

[53] Plankar M., Brežan S., Jerman I. (2013). The principle of coherence in multi-level brain information processing. Prog. Biophys. Mol. Biol..

[54] Duncan J. (1984). Selective attention and the organization of visual information. J. Exp. Psychol. Gen..

[55] O’Craven K.M., Downing P.E., Kanwisher N. (1999). fMRI evidence for objects as the units of attentional selection. Nature.

[56] Rizkallah J., Benquet P., Kabbara A., Dufor O., Wendling F., Hassan M. (2018). Dynamic reshaping of functional brain networks during visual object recognition. J. Neural Eng..

[57] Gorantla V.R., Tedesco S., Chandanathil M., Maity S., Bond V., Lewis C., Millis R.M. (2020). Associations of alpha and beta interhemispheric EEG coherences with indices of attentional control and academic performance. Behav. Neurol..

[58] Aydın S., Demirtaş S., Yetkin S. (2018). Cortical correlations in wavelet domain for estimation of emotional dysfunctions. Neural Comput. Appl..

